# The gE/gI complex is necessary for kinesin-1 recruitment during alphaherpesvirus egress from neurons

**DOI:** 10.1128/jvi.01650-24

**Published:** 2024-12-09

**Authors:** Drishya Diwaker, DongHo Kim, Dylann Cordova-Martinez, Nivedita Pujari, Bryen A. Jordan, Gregory A. Smith, Duncan W. Wilson

**Affiliations:** 1 Department of Developmental and Molecular Biology, Albert Einstein College of Medicine 2006 , New York, New York, USA; 2 Department of Microbiology-Immunology, Northwestern University Feinberg School of Medicine 547641 , Chicago, Illinois, USA; 3 Dominick P. Purpura Department of Neuroscience, Albert Einstein College of Medicine 200752 , New York, New York, USA; The University of Arizona, Tucson, Arizona, USA

**Keywords:** alphaherpesviruses, anterograde transport, herpes simplex virus, pseudorabies virus, axonal transport

## Abstract

Following reactivation of a latent alphaherpesvirus infection, viral particles are assembled in neuronal cell bodies, trafficked anterogradely within axons to nerve termini, and spread to adjacent epithelial cells. The virally encoded membrane proteins US9p and the glycoprotein heterodimer gE/gI of pseudorabies virus (PRV) and herpes simplex virus type 1 (HSV-1) play critical roles in anterograde spread, likely as a tripartite gE/gI-US9p complex. Two kinesin motors, kinesin-1 and kinesin-3, are implicated in the egress of these viruses, but how gE/gI-US9p coordinates their activities is poorly understood. Here, we report that PRV, in addition to associating with the kinesin-3 motor KIF1A, recruits the neuronal kinesin-1 isoforms KIF5A and KIF5C, but not the broadly expressed isoform KIF5B, during egress from differentiated CAD neurons. Similarly, in the axons of dorsal root ganglia (DRG)-derived sensory neurons, PRV colocalized with KIF5C but not KIF5B. In differentiated CAD cells, the association of KIF1A with egressing PRV was dependent upon US9p, whereas the recruitment of KIF5 isoforms required gE/gI. Consistent with these findings, the number of PRV particles trafficking within CAD neurites and the axons of DRG neurons increased when kinesin-1 motor activity was upregulated by hyperacetylating microtubules using trichostatin A (TSA) or tubacin, and this enhanced trafficking depended upon the presence of gE/gI. We propose that, following its recruitment by US9p, KIF1A delivers PRV particles to a location where KIF5 motors are subsequently added by a gE/gI-dependent mechanism. KIF5A/C isoforms then serve to traffic viral particles along axons, resulting in characteristic recrudescent infection.

**IMPORTANCE:**

Alphaherpesviruses include important human and veterinary pathogens that share a unique propensity to establish life-long latent infections in the peripheral nervous system. Upon reactivation, these viruses navigate back to body surfaces and transmit to new hosts. In this study, we demonstrate that the virus gE/gI-US9p membrane complex routes virus particles down this complex neuronal egress pathway by coordinating their association with multiple kinesin microtubule motors.

## INTRODUCTION

During infection of neurons by alphaherpesviruses such as herpes simplex virus type 1 (HSV-1) and pseudorabies virus (PRV), capsids are assembled and packaged with the viral double-stranded DNA (dsDNA) genome in the nucleoplasm. These capsids then exit the nucleus, traverse the cytoplasm, and bud into the lumen of cytoplasmic organelles, generating an infectious enveloped virion contained within a carrier vesicle. Vesicle-associated enveloped viral particles subsequently traffic from the soma into and along axons, eventually reaching distal terminals where they are released by non-lytic exocytosis to infect innervated tissues (1–7). In addition, non-enveloped capsids may also traffic from the soma into and along axons in some types of neuron, eventually enveloping at the nerve terminal (8–12). The transport of viral particles within the neuronal cell body, and along axons, is dependent upon kinesin microtubule motors (2, 13–15). Of the 45 known kinesins in the mammalian genome (16–19), alphaherpesviruses preferentially use members of the kinesin-1 and kinesin-3 families (2, 15, 20–23).

Kinesin-3 motors are monomers when inactive, but their association with the lipid bilayers of vesicular cargo drives dimerization and activation (18, 24). Dimeric kinesin-3 motors are fast (~1.0–2.5 µm s^−1^) and exhibit “superprocessive” motion with average run lengths of ∼10 µm: nearly 10 times that of kinesin-1 motors (24). KIF1A, a kinesin-3 motor that is abundantly expressed in the brain, drives long-distance axonal transport of synaptic vesicle precursors from neuronal cell bodies to nerve terminals (16, 17, 19). PRV recruits KIF1A via three virally encoded membrane proteins (2, 3, 15, 25–29). US9p is a small, non-glycosylated lipid raft-associated type II membrane protein, while glycoproteins E and I (gE and gI) are type I membrane proteins that function as a heterodimer (2, 7, 25, 30–32). For HSV-1 and PRV, gE/gI and US9p promote anterograde spread within neurons and in the nervous system (7, 29–39). Biochemical studies indicate that gE/gI and US9p form a tripartite complex within detergent-resistant membrane (DRM) “lipid rafts” in PRV-infected neurons, and the presence of gE/gI and US9p is sufficient to recruit KIF1A into DRMs (40). PRV US9p coimmunoprecipitates with KIF1A in detergent extracts prepared from PRV-infected PC12 cells (21), and gE/gI stimulates this interaction (41). When egressing membrane-associated PRV particles are isolated from the cytoplasm of infected cells, they are found in association with KIF1A, but not when gE/gI and US9p are absent. Furthermore, these gE/gI-US9p-null viral particles exhibit substantially reduced plus end-directed motion along microtubules in a cell-free trafficking assay, together with an increased likelihood of minus end-directed motion and more frequent reversals in direction (20). Together, these data suggest that one function of the gE/gI-US9p complex is to recruit KIF1A for transport of alphaherpesvirus particles. Consistent with this model, the axonal sorting, transport, and anterograde spread defect of gE/gI-US9p null PRV virions can be largely corrected (or at least suppressed) by directing the recruitment of KIF1A to PRV particles by artificial means (40). In addition, gE/gI and US9p may contribute to the efficient anterograde spread of alphaherpesviruses by promoting the efficiency of assembly of HSV-1 and PRV particles in the soma (5, 7, 42, 43)

Although anterograde-trafficking PRV and HSV-1 particles co-transport with KIF1A in axons (21, 40), the primary role of US9p-recruited KIF1A appears to be during a prerequisite step that routes viral particles in the neuronal cell body to the axon. In support of this, very few US9p-null PRV particles reach the axon during egress, but those that do exhibit microtubule-based anterograde transport kinetics are indistinguishable from wild type (44). These data raise the question of which kinesin motors are critical for PRV and HSV-1 transport within the axon, and how these motors are recruited to viral particles.

Kinesin-1 motors are heterotetramers consisting of two motor-containing kinesin heavy chains (KHC) and two kinesin light chains (KLC) (15–19). There are three KHC isoforms, which when complexed with KLCs form the KIF5A, KIF5B, and KIF5C motors. KIF5B is ubiquitously expressed while KIF5A and KIF5C are largely neuronal (15–19). Each kinesin-1 motor always contains two identical KIF5 KHCs (18). In axons, kinesin-1 preferentially traffics along acetylated microtubules, which are commonly non-dynamic, stabilized, and bundled (45–47). Kinesin-1 is therefore well suited for microtubule-directed anterograde traffic along axons and transports a variety of cargo including mitochondria, lysosomes, and synaptic vesicle precursors (16, 17). The role of kinesin-1 in neurons has been modeled using the mouse catecholaminergic cell line CAD, both for the study of alphaherpesvirus (7, 12, 20, 22) and endogenous neuronal cargo transport (47, 48). Upon differentiation, CAD neurons express multiple neuronal markers and develop long neurites analogous to axons (49–51). In HSV-1-infected CAD neurons, egressing viral particles colocalize with KIF5C in the neurite (22), and RNA interference (RNAi)-mediated silencing of KIF5A/B/C or KLCs (16–18) impairs their transport (22). These findings are substantiated by immunogold electron microscopy in primary neurons showing the localization of kinesin-1 motors to viral particles in axons (52). During PRV replication in epithelial cells, kinesin-1 motors bind to the large inner tegument protein UL36p, become “assimilated” into the interior of enveloped viral particles, and promote capsid transport to the nucleus during subsequent infection of neurons (23, 53). However, it is unknown whether UL36p-mediated assimilation is related to the mechanism by which kinesin-1 motors associate with the cytoplasmic face of alphaherpesvirus-bearing organelles during anterograde transport in neurons, and a number of other virally encoded proteins have been proposed as kinesin-1 binding partners (15, 54, 55).

Previously, we reported that PRV particles egressing from differentiated CAD neural cells associate with either KIF1A (kinesin-3) or KIF5C (a neuronal-specific isoform of kinesin-1), but rarely with both motors simultaneously (20). Furthermore, PRV particles were associated with KIF1A irrespective of the CAD differentiation state but bound to KIF5C only in differentiated CAD neurons, despite differentiated and undifferentiated CAD cells expressing equivalent amounts of KIF5C protein (20). Loss of the gE/gI-US9p complex reduced the efficiency with which PRV particles recruited both KIF1A and KIF5C motors and compromised their ability to undergo plus end-directed transport along microtubules *in vitro* (20). The goals of the current study were to test whether PRV exhibits a preference for neuron-specific isoforms of kinesin-1 in differentiated CAD neurons and primary sensory neurons and to determine the individual roles of US9p and gE/gI in PRV association with these various kinesin motors. Finally, we examined whether PRV particles undergoing egress in the axons of differentiated CAD neurons and primary sensory neurons behave like KIF1A- or kinesin-1-borne cargo by testing their response to microtubule hyperacetylation.

## RESULTS

### PRV preferentially recruits neuron-specific isoforms of kinesin-1 in differentiated CAD cells

PRV-GS4284 is a derivative of the PRV-Becker strain that expresses a mCherry-UL25p capsid fusion protein and is referred to as the “P” (Parental) strain throughout this study (56). We previously demonstrated that P PRV recruits the neuron-specific kinesin-1 motor KIF5C during egress from neural CAD cells, but only if the CAD cells were neuronally differentiated (20). In the current study, we first tested whether PRV also recruits the other neuronal-specific kinesin-1 isoform KIF5A and the broadly expressed isoform KIF5B (57–59) and examined how the binding of those motors is affected by the differentiation state of the CAD cell. We prepared undifferentiated CAD cells or differentiated CAD neurons and infected them with P PRV. After 20 h, we collected whole-cell lysates for western blotting to compare KIF5 isoform protein levels and also prepared a post-nuclear supernatant (PNS). The PNS was subjected to density gradient centrifugation to isolate a membrane-associated buoyant “float-up” fraction enriched in egressing viral particles, as previously described (20). Organelles in the float-up gradient fraction were attached to glass coverslips, fixed, immunostained for KIF5 isoforms, and the frequency of mCherry-UL25p capsid/anti-motor fluorescence colocalization was determined.

**Fig 1:**
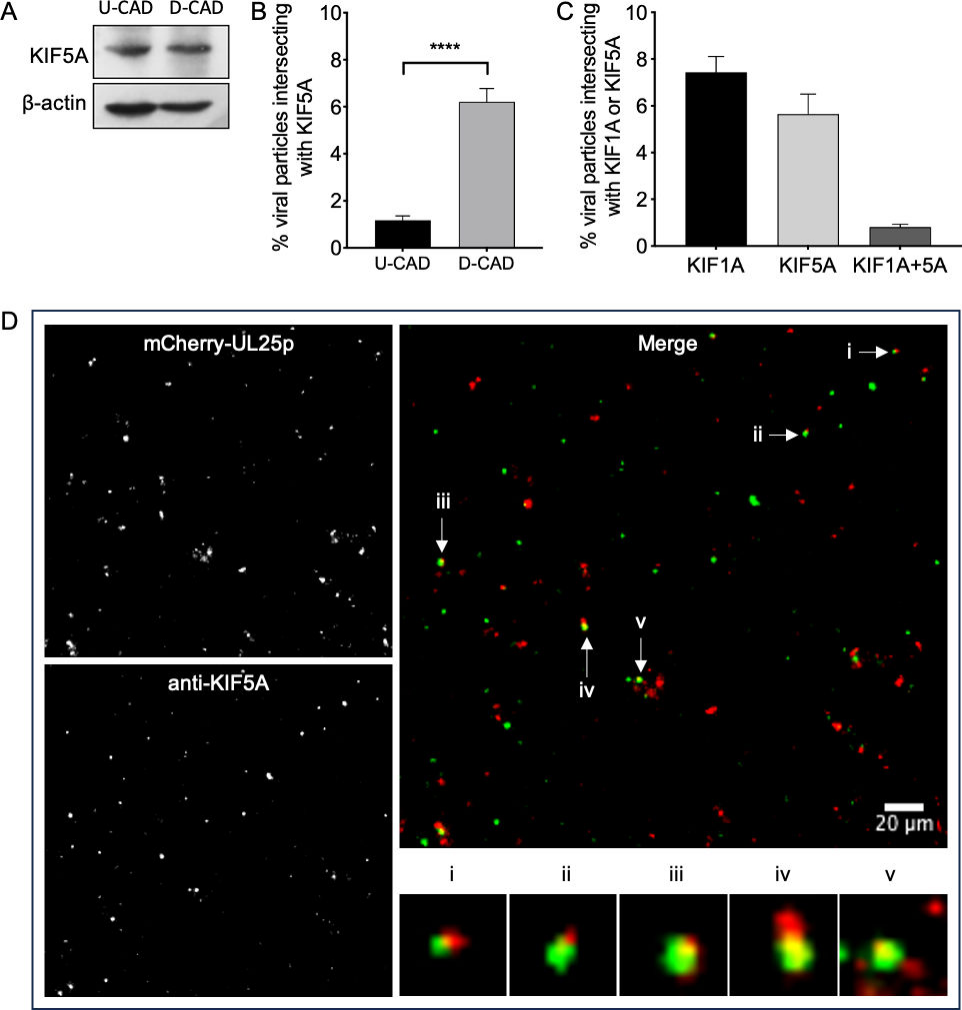
PRV recruits KIF5A and KIF1A to distinct populations of virus particles in differentiated CAD neurons. (**A**) Whole-cell lysates were prepared from equivalent numbers of P PRV-infected, undifferentiated CAD cells or differentiated CAD neurons (U-CAD and D-CAD, respectively) and then western blotted for KIF5A or β-actin as a loading control, as indicated at left. (**B**) Float-up fractions were prepared from P PRV-infected U-CAD and D-CAD cells, immunostained for KIF5A, and imaged for mCherry-tagged capsid fluorescence (red channel) and anti-KIF5A (blue channel). Red particles were scored for colocalization with KIF5A and plotted as a percent of total red particles in the field. Plotted values are mean and SD from the mean for 15,438 particles (U-CAD) and 17,321 particles (D-CAD), in each case counted from at least 15 microscopic fields. (**C**) Undifferentiated CAD cells were transfected to express mCitrine-KIF1A, differentiated, and infected with P PRV. Float-up fractions were prepared and immunostained for KIF5A then fields simultaneously imaged for mCherry-tagged capsids (red channel), mCitrine-KIF1A (green channel), and anti-KIF5A (blue channel). Red particles were scored for colocalization with mCitrine-KIF1A, KIF5A, or both motors simultaneously, as indicated on the X axis, and colocalization plotted as a percent of total red particles in the field. Plotted values are mean and SD from the mean for 27,995 particles counted from 25 microscopic fields. ****P ≤ 0.0001. (**D**) A microscopic field representative of those used to generate the data in (**B**). The red channel (mCherry-UL25p) and blue channel (anti-KIF5A) are shown in grayscale on the left, and a merged colored image are shown on the right (the anti-KIF5A image is pseudocolored in green). Merged panel scale bar: 20 µm. Five mCherry-UL25p fluorescing puncta that also exhibit anti-KIF5A fluorescence are indicated by arrows and labeled i–v. They are also shown at 7× higher magnification in the gallery below the merged field.

The data for KIF5A are shown in Fig. 1A through C, and a representative microscopic field is shown in Fig. 1D. We found that KIF5A protein levels were similar in differentiated CAD neurons and undifferentiated CAD cells (Fig. 1A), but PRV particles were more frequently associated with KIF5A motors when isolated from differentiated, rather than undifferentiated CAD cells (Fig. 1B). We also tested whether KIF5A was associated with a population of PRV particles distinct from those bound to the kinesin-3 motor KIF1A, as is the case for KIF5C (20). To simultaneously detect KIF5A and KIF1A, we combined immunofluorescent detection of KIF5A with transient expression of KIF1A fused to the mCitrine fluorescent protein, as previously described (20, 60, 61). CAD cells were transfected to express mCitrine-KIF1A, differentiated, and then infected with PRV. As shown in Fig. 1C, distinct subpopulations of PRV particles were associated with either KIF1A or KIF5A, but rarely both motors at the same time. Grayscale and merged color images of a representative anti-KIF5A stained microscopic field and a gallery of PRV capsid-associated structures exhibiting KIF5A immunoreactivity are shown in Fig. 1D. Typically, 5%–15% of P (parental) PRV capsids in our extracts colocalized with KIF1A or KIF5 motors (Fig. 1 and data presented below), as seen in our earlier study (20). We note that this gradient-purified subcellular fraction includes all membrane-associated capsids present in the cell cytoplasm at the time of extract preparation, and thus represents capsids at all stages of virus assembly and egress. The comparatively low proportion of kinesin-associated capsids may reflect the distinct subpopulation that is actively undergoing motor-dependent transport at the time of cell breakage. Furthermore, the efficiency with which mCitrine-KIF1A associates with egressing virus might be reduced by competition with endogenous non-fluorescent KIF1A, or because the mCitrine fluorescent protein causes steric interference with the apparatus responsible for motor recruitment onto viral particles.

**Fig 2:**
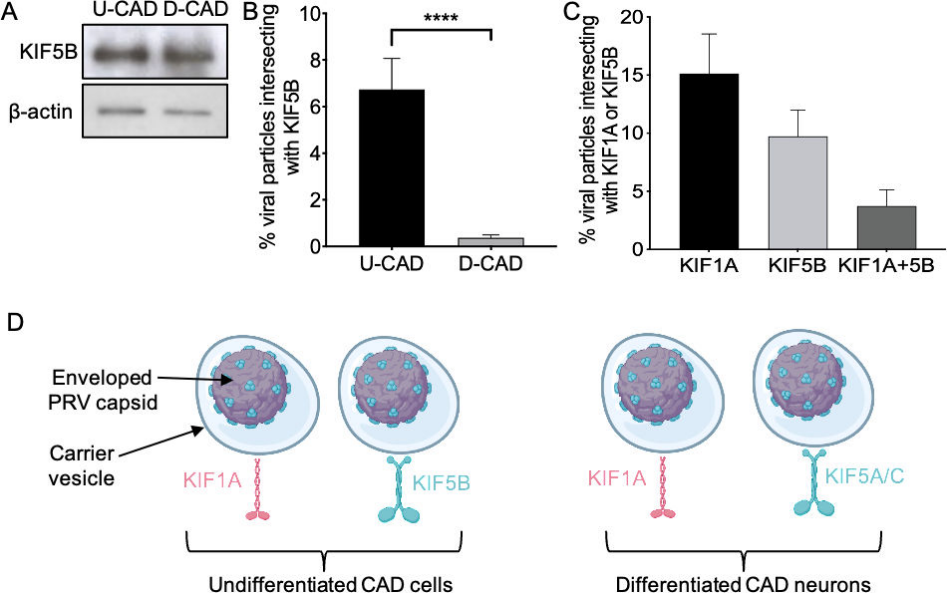
PRV recruits KIF5B and KIF1A to distinct populations of virus particles in undifferentiated CAD cells. (**A**) Whole-cell lysates were prepared from equivalent numbers of P PRV-infected U-CAD and D-CAD cells and then western blotted for KIF5B or β-actin as a loading control, as indicated at left. (**B**) Float-up fractions were prepared from P PRV-infected U-CAD and D-CAD cells, immunostained for KIF5B, and imaged for mCherry-tagged capsid fluorescence (red channel) and anti-KIF5B (blue channel). Red particles were scored for colocalization with KIF5B and plotted as a percent of total red particles in the field. Plotted values are mean and SD from the mean for 15,143 particles (U-CAD) and 28,002 particles (D-CAD) in each case counted from at least seven microscopic fields. (**C**) Undifferentiated CAD cells were transfected to express mCitrine-KIF1A and infected with P PRV. Float-up fractions were immunostained for KIF5B, and fields were simultaneously imaged for mCherry-tagged capsids (red channel), mCitrine-KIF1A (green channel), and anti-KIF5B (blue channel). Red particles were scored for colocalization with KIF1A, KIF5B, or both motors simultaneously, as indicated on the X axis, and colocalization plotted as a percent of total red particles in the field. Plotted values are mean and SD from the mean for 7,344 particles counted from five microscopic fields. (**D**) Schematic summarizing the data in Fig. 1 and 2. One or more enveloped PRV capsids reside within the bounding lipid envelope of a carrier vesicle, as indicated. In undifferentiated CAD cells (left), PRV recruits KIF1A to one population of carrier vesicles and the ubiquitous kinesin-1 KIF5B to another. Rarely are both motors observed on the same viral particle. A similar situation is seen in differentiated CAD neurons (right), except that PRV selects the neuron-specific kinesin-1 motors KIF5A and KIF5C, rather than KIF5B.

Our findings for KIF5B are shown in Fig. 2A through C. We found that levels of KIF5B protein expression were equivalent in undifferentiated CAD cells and differentiated CAD neurons (Fig. 2A). Nevertheless, in direct contrast to KIF5A and KIF5C, KIF5B only associated with PRV particles in undifferentiated CAD cells and not differentiated CAD neurons (Fig. 2B). PRV particles also recruited KIF1A in undifferentiated CAD cells, and as seen for KIF5A and KIF5C in differentiated CAD neurons ([20] and Fig. 1C), KIF5B and KIF1A motors were rarely found together on the same viral particle (Fig. 2C). The data for KIF1A, KIF5B, and KIF5A/C binding are summarized schematically in Fig. 2D.

### US9p and gE/gI play distinct roles in the recruitment of KIF1A and KIF5 to PRV particles

We previously reported that the deletion of the three genes encoding the gE/gI-US9p complex reduced the frequency and processivity of plus end-directed viral transport along microtubules *in vitro* and resulted in loss of both KIF1A and KIF5C motors from egressing PRV particles (20). We next tested whether gE/gI and US9p play distinct roles in the recruitment of KIF1A and KIF5 kinesins to PRV. We used strains isogenic to the P strain PRV-GS4284, but encoding a deletion of the US9 gene (44), or a deletion spanning US7 (encoding gE) and most of US8 (encoding gI). These strains, PRV-GS5469 and PRV-GS7453, are here referred to as ΔUS9p and ΔgE/gI respectively.

**Fig 3:**
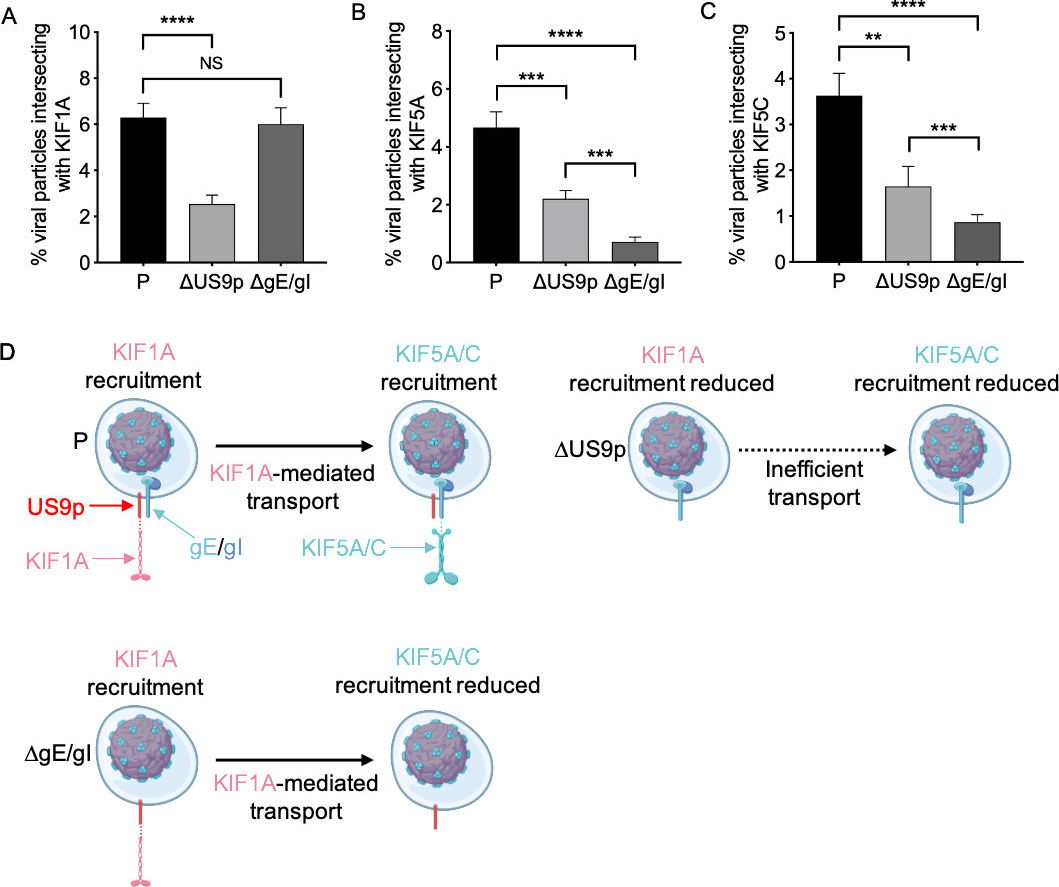
The consequences of US9p or gE/gI loss for recruitment of kinesins KIF1A, KIF5A, and KIF5C in differentiated CAD neurons. (**A**) Undifferentiated CAD cells were transfected to express mCitrine-KIF1A, differentiated, and infected with PRV strains P,ΔUS9p or ΔgE/gI, as indicated. Float-up fractions were prepared and red particles scored for colocalization with KIF1A (green channel) as in Fig. 1C. Colocalization is plotted as a percent of total red particles in the field. Plotted values are mean and SD from the mean for 61,923 P, 28,424 ΔUS9p, and 34,457 ΔgE/gI particles counted from 50, 43, and 48 microscopic fields, respectively. (**B**) Similar to (**A**) but float-up fractions were immunostained for KIF5A. Colocalization between red particles and KIF5A (blue channel) was plotted as a percent of total red particles in the field. Plotted values are mean and SD from the mean for 24,093 P, 24,472 ΔUS9p, and 23,418 ΔgE/gI particles, each counted from 18 microscopic fields. (**C**) Similar to (**A**) but float-up fractions were immunostained for KIF5C. Colocalization between red particles and KIF5C (blue channel) was plotted as a percent of total red particles in the field. Plotted values are mean and SD from the mean for 34,377 P, 30,216 ΔUS9p, and 41,692 ΔgE/gI particles, counted from 26, 25, and 29 microscopic fields, respectively. NS: not significant, **P ≤ 0.01 and ***P ≤ 0.001. (**D**) Schematic to summarize and explain these data. Egressing viral particles are represented as in Fig. 2D. The panel shows the predicted structure and properties of the P (upper left), ΔgE/gI (lower left), and ΔUS9p (upper right) strains of PRV. For the P virus, the bounding lipid bilayer of the carrier vesicle contains an intact gE/gI-US9p complex (represented by a red bar and blue lollipops, respectively). The deletion mutant viruses lack either US9p or gE/gI, as indicated, and the consequences of this for trafficking and kinesin recruitment are depicted. US9p and gE/gI are connected to the kinesins by broken lines to emphasize that the interaction between gE/gI-US9p and the motor proteins may not be direct. See text for more details.

We infected differentiated CAD neurons with the P, ΔUS9p, or ΔgE/gI strains and, using a similar approach to that in Fig. 1 and 2, examined egressing viral particles present in a gradient-isolated subcellular fraction for the presence of KIF1A and KIF5 motors. We found that the deletion of US9p significantly reduced the association of viral particles with KIF1A, KIF5A, and KIF5C (Fig. 3). The loss of KIF1A (Fig. 3A) in the absence of US9p is consistent with previous biochemical data (21, 40, 41) and resembles the results seen following the removal of the entire gE/gI-US9p complex (20). The effect of US9p loss upon KIF5A and KIF5C recruitment, however, was unexpected (Fig. 3B and C). These data could indicate that US9p recruits not only KIF1A but also KIF5 motors. Alternatively, KIF1A recruitment by US9p might be a prerequisite for KIF5 association, perhaps by serving to deliver PRV particles to an intracellular location where KIF5 motors subsequently become attached (see Fig. 3D and Discussion for more details).

We next tested the effect of the removal of the gE/gI heterodimer from the gE/gI-US9p complex. The loss of gE/gI had no effect upon KIF1A recruitment (Fig. 3A) but caused reductions in KIF5A/C association that were significantly more severe than seen by the absence of US9p (Fig. 3B and C). We conclude from these data that, in differentiated CAD neurons, gE/gI serves as the critical KIF5 recruitment factor. We summarize and interpret these data in Fig. 3D. The P strain of PRV (Fig. 3D, top left of panel) expresses a functional gE/gI-US9p complex in the bounding lipid bilayer of its carrier vesicle. During egress from the soma, viral particles recruit KIF1A (pink kinesin) by a mechanism dependent upon US9p (red bar). This bound KIF1A ensures virus particle transport to a location where KIF1A is removed, and in differentiated CAD neurons, the KIF5A and KIF5C isoforms of kinesin-1 (blue kinesins) are attached in a process that requires gE/gI (blue lollipops). The ΔgE/gI strain of PRV (Fig. 3D, lower left of panel) recruits KIF1A normally and presumably traffics normally to the location where KIF5A/C is to be recruited, but KIF5A/C binding fails in the absence of gE/gI. Finally, the ΔUS9p strain of PRV (Fig. 3D, upper right of panel) is reduced in its ability to recruit KIF1A, and diminished numbers of viral particles traffic to the location where gE/gI-dependent KIF5A/C motor association occurs.

**Fig 4:**
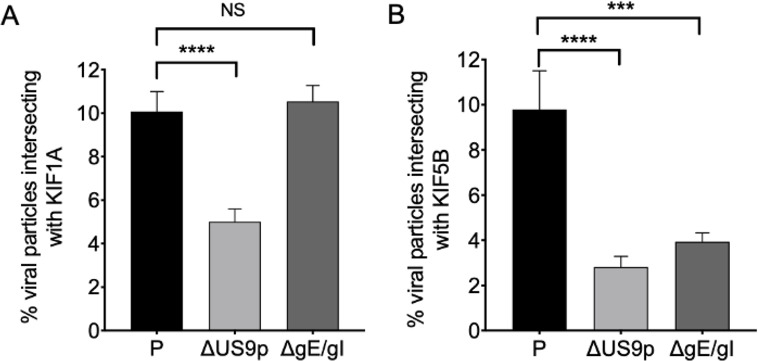
The consequences of US9p or gE/gI loss for recruitment of kinesins KIF1A and KIF5B in undifferentiated CAD cells. (**A**) Undifferentiated CAD cells were transfected to express mCitrine-KIF1A then infected with PRV strain P,ΔUS9p or ΔgE/gI, as indicated. Float-up fractions were prepared and red particles scored for colocalization with KIF1A (green channel) as in Fig. 1C. Colocalization is plotted as a percent of total red particles in the field. Plotted values are mean and SD from the mean for 21,514 P, 20,735 ΔUS9p, and 23,805 ΔgE/gI particles, counted from 19, 18, and 22 microscopic fields, respectively. (**B**) Similar to (**A**) but float-up fractions were immunostained for KIF5B. Colocalization between red particles and KIF5B (blue channel) was plotted as a percent of total red particles in the field. Plotted values are mean and SD from the mean for 20,586 P, 27,202 ΔUS9p, and 29,171 ΔgE/gI particles, counted from 17, 23, and 29 microscopic fields, respectively.

We performed similar experiments in undifferentiated CAD cells (Fig. 4). Deletion of US9p diminished the ability of PRV particles to recruit both KIF1A and KIF5B (Fig. 4A and B), while loss of gE/gI only reduced KIF5B recruitment (Fig. 4A and B). These data can be explained if US9p-dependent KIF1A transport is required for subsequent gE/gI-dependent attachment of kinesin KIF5B, similar to the model depicted in Fig. 3D. If correct, this model implies that KIF1A-mediated transport is needed for subsequent KIF5B recruitment even though these undifferentiated CAD cells lack neurites and other differentiation-dependent features (47–51).

### PRV preferentially recruits KIF5C over KIF5B or KIF1A in the axons of DRG-derived primary sensory neurons

**Fig 5:**
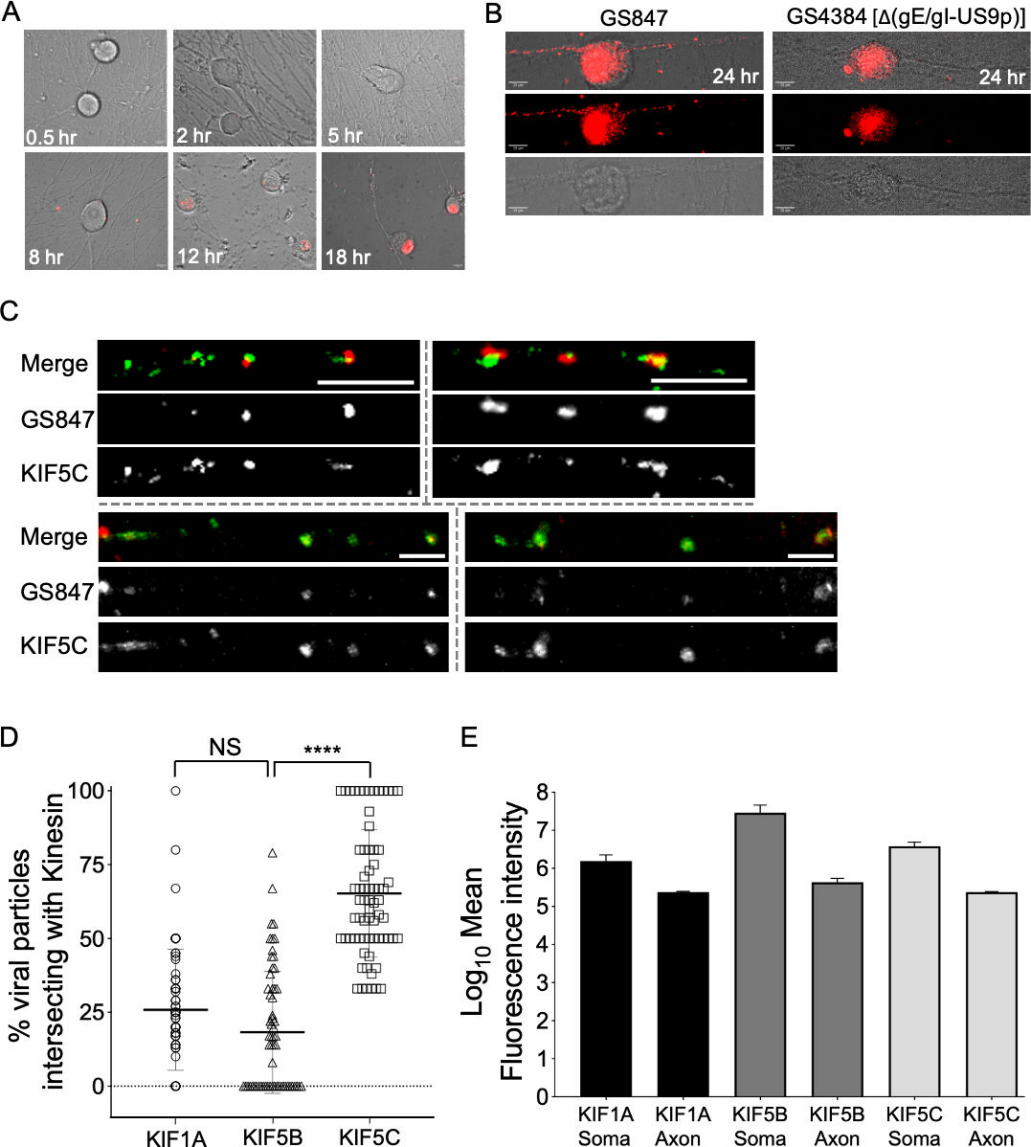
PRV preferentially recruits KIF5C over KIF5B or KIF1A in the axons of DRG-derived primary sensory neurons. (**A**) Rat embryonic DRG-derived neurons were infected by PRV strain GS847, fixed between 0.5 and 18 h p.i. as indicated, then representative brightfield and red channel (mRFP1-capsid fluorescence) images merged. (**B**) Similar to (**A**) except neurons were infected by GS847 (left column) or its Δ(gE/gI-US9p)-derivative GS4384 (right column) then fixed at 24 h p.i. Infected neurons were imaged in brightfield (bottom row), for mRFP1-capsid fluorescence (middle row) or merged (top row). Scale bar: 10 µm. (**C**) DRG-derived neurons were infected by GS847, fixed at 18 h p.i., and immunostained for KIF5C. Each group of three images shows a representative length of infected axon imaged for anti-KIF5C fluorescence (green channel, bottom), GS847 mRFP1-capsid fluorescence (red channel, middle), or merged (top). Scale bar: 5 µm. Broken gray lines separates images collected from four individual axons. (**D**) DRG-derived neurons were infected exactly as in (**C**) and immunostained for KIF5B, or KIF5C, or imaged for mCitrine-KIF1A fluorescence expressed following infection by a recombinant lentivirus. Numbers of red-fluorescent capsid-associated axonal puncta colocalizing with each kinesin in a 32 mm length of infected axon were plotted as a percent of total puncta. Plotted values are the mean and SD from the mean for a total of 592 (mCitrine-KIF1A), 573 (anti-KIF5B), and 486 (anti-KIF5C) viral puncta, in all cases imaged in at least 20 axons in each of three independent experiments. Statistical significance was determined by one-way analysis of variance (ANOVA) with a post hoc Tukey test. (**E**) Identical experiment to (**D**) except that the intensity of mCitrine-KIF1A, anti-KIF5B, or anti-KIF5C fluorescence was measured in the soma and a 23 mm length of proximal axon. Plotted values are Log_10_ of the mean, and SD from the mean, for the sum of fluorescence intensity values recorded from the following numbers of soma and their proximal axons (soma/axons): mCitrine-KIF1A (8/16), KIF5B (10/20), and KIF5C (10/20).

Although CAD cells are widely used as a model system for the study of alphaherpesvirus assembly and egress (7, 12, 20, 22, 62, 63), we wished to next investigate PRV transport and kinesin selection in the axons of primary sensory neurons, a more natural host environment. Rat embryonic dorsal root ganglia (DRG) neurons were isolated, cultured *in vitro*, and then infected with PRV. In some experiments, they were also infected with a recombinant lentivirus designed to express the mCitrine-KIF1A fusion protein described above (Fig. 1 to 4 and [20]). In these experiments, we used PRV strain GS847, which expresses an mRFP1 (monomeric red fluorescent protein 1)-UL35p capsid fusion protein (64) and which we found easier to image in the axons of DRG neurons than the PRV strain P, used in our CAD studies. Under our conditions, mRFP1-UL35p fluorescent puncta became visible in DRG axons between 18 and 24 h post infection (p.i.; Fig. 5A and B). Most of these PRV puncta depended upon gE/gI and/or US9p for their appearance in axons, as expected (1, 2, 28, 32, 39), since their numbers were 80% lower in neurons infected by PRV-GS4384, a strain identical to PRV-GS847 but lacking the genes encoding gE, gI, and US9p (Fig. 5B, quantitated in Fig. 8).

We next examined the degree to which PRV fluorescent puncta colocalized with lentivirus-expressed mCitrine-KIF1A fluorescence and anti-KIF5B or anti-KIF5C immunofluorescence in axons. Representative images of PRV/KIF5C colocalization in segments of proximal axon are shown in Fig. 5C, and colocalization with all three kinesins is quantitated in Fig. 5D. We found that axonal PRV particles frequently colocalized with KIF5C, but only a small minority appeared to be associated with mCitrine-KIF1A or KIF5B. Between 50% and 100% of the PRV particles colocalized with KIF5C in four-fifths (~82%) of the axon segments examined, and in no axons did the frequency of PRV/KIF5C colocalization fall below 33%. Conversely, when immunostained for KIF5B, two-thirds (68%) of axon segments exhibited a frequency of PRV/KIF5B colocalization below 26%, and nearly half of the axon segments (44%) contained no KIF5B-labeled viral puncta at all (Fig. 5D). The mean frequency with which mCitrine-KIF1A colocalized with PRV resembled that for KIF5B, but the distribution was broader, and almost all axon segments contained some mCitrine-KIF1A-labeled PRV puncta (Fig. 5D). The observed frequency of PRV colocalization with each motor was not related to the sensitivity of motor detection since the sum of the intensities of mCitrine-KIF1A fluorescence or anti-KIF5B immunofluorescence was each similar to, or greater than, anti-KIF5C immunofluorescence in axons (Fig. 5E). These data show that PRV preferentially recruits kinesin-1, rather than kinesin-3 (KIF1A), in the axons of these neurons, and favors the neuronal-specific kinesin-1 isoform KIF5C over KIF5B.

### PRV localization to CAD neurites and the axons of sensory neurons is stimulated by microtubule hyperacetylation

**Fig 6:**
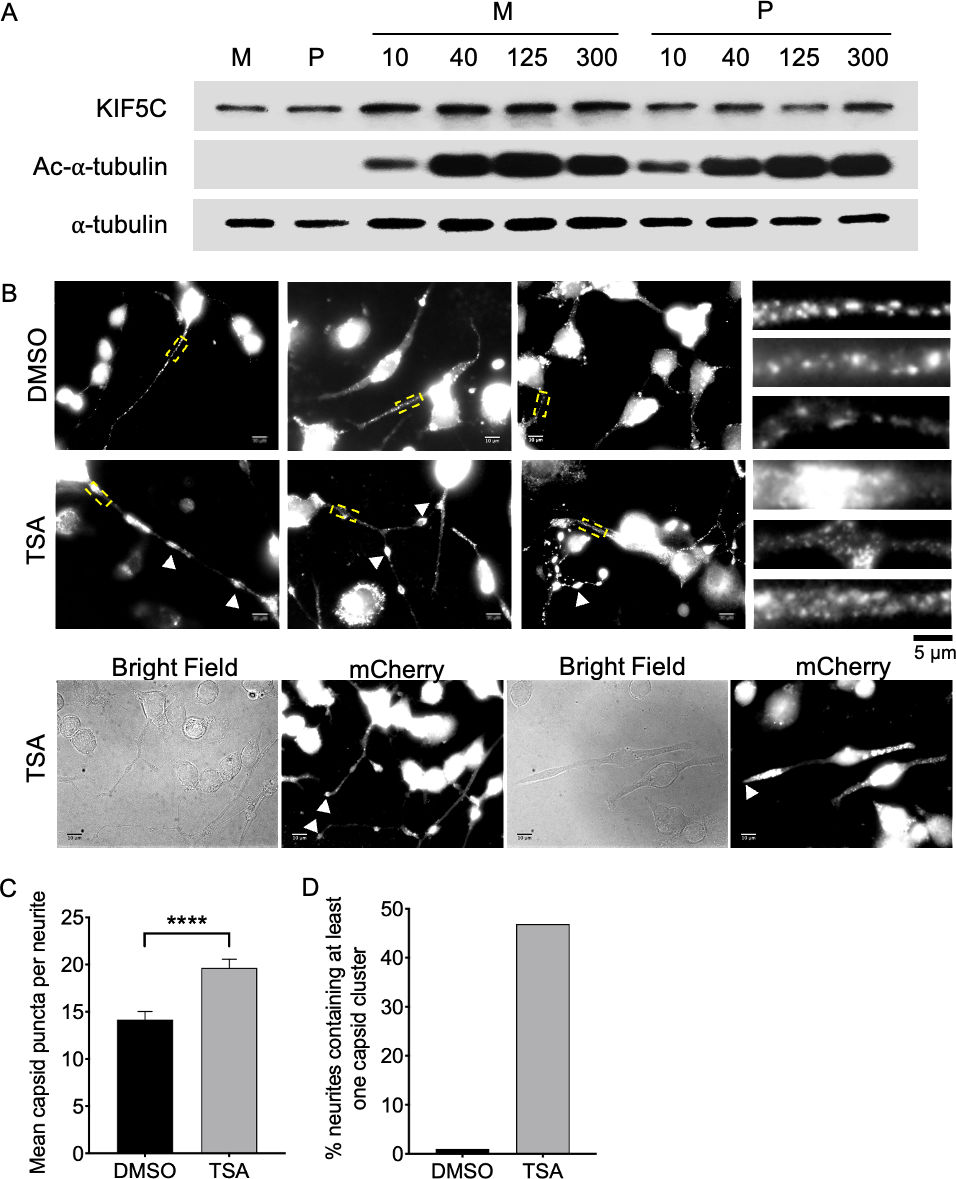
Effect of TSA on microtubule acetylation and PRV localization to CAD neurites. (**A**) Differentiated CAD neurons were infected with parental PRV (**P**) or mock infected (**M**) then incubated for 24 h in the absence of TSA (two left-hand lanes), or in the presence of 10, 40, 125, or 300 nM TSA, as indicated at top. Whole-cell lysates were prepared and western blotted for KIF5C, acetylated (AC)-α-tubulin or total α-tubulin, as indicated at left. (**B**) Top and middle row of images: P PRV-infected, differentiated CAD neurons were incubated with 125 nM TSA or dimethylsulfoxide (DMSO) solvent control (as indicated at left) for 24 h then fixed and imaged for mCherry-capsid fluorescence. Cell body fluorescence is deliberately overexposed in order to image particles in neurites. Regions boxed in broken yellow lines are magnified 5× at the right of each row, with those from the left, middle, and right panels depicted at top, middle, and bottom, respectively. White arrowheads indicate large viral particle clusters along the shafts of neurites. Bottom row of images: Identical experimental conditions to the middle row. Left pair of images and right pair of images represent two microscopic fields, each imaged for mCherry-capsid fluorescence or in bright field, as indicated. Arrowheads in the mCherry fields point to viral particle accumulations at the tips of neurites. Scale bars are 10 and 5 µm, as indicated. (**C**) Images similar to those in (**B**) were used to count numbers of individual capsid-associated viral particle puncta (other than those in clusters) present in infected neurites in DMSO- or TSA-treated CAD neurons. Plotted values are mean and SD from the mean for numbers of puncta counted in the neurites of 59 DMSO- and 57 TSA-treated, infected CAD neurons. (**D**) Images similar to those in (**B**) were used to determine the percentage of infected CAD neurons that contained at least one large capsid-associated cluster in their neurites, following DMSO or TSA treatment (99 and 96 infected CAD cell neurites examined, respectively).

**Fig 7:**
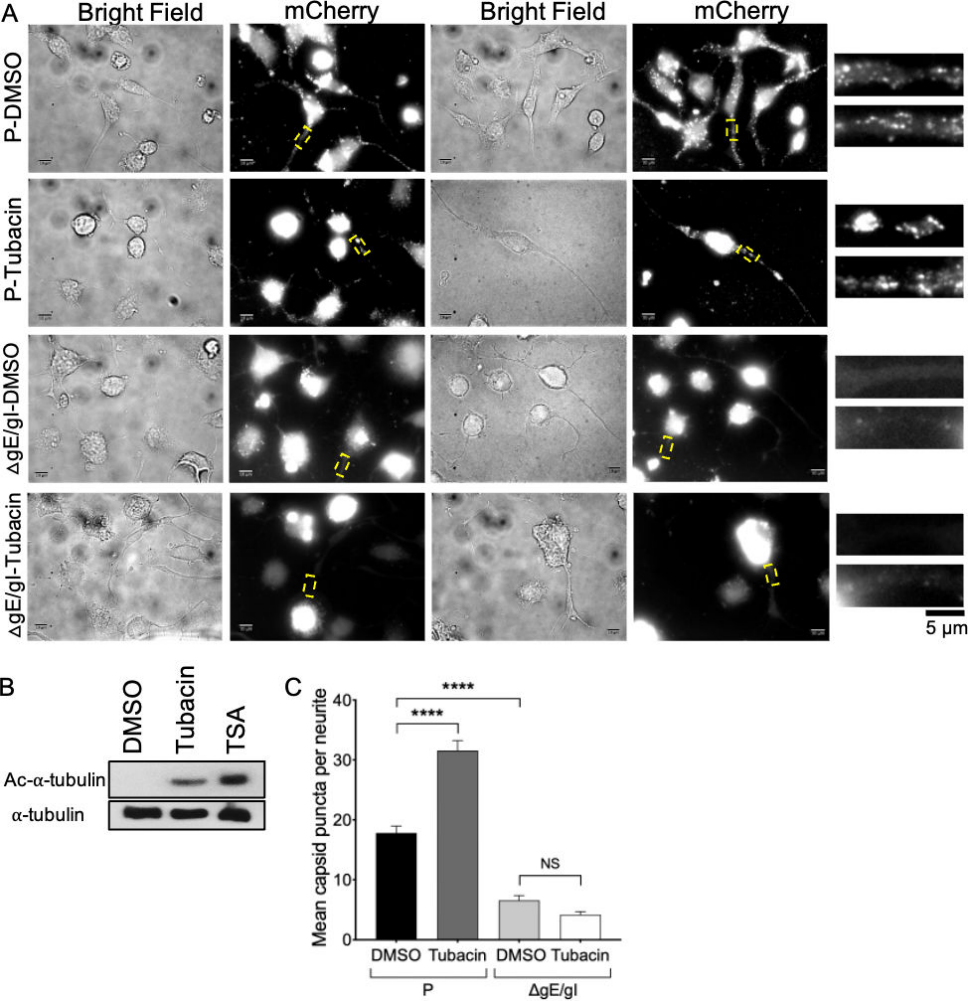
Tubacin stimulates microtubule acetylation and gE/gI-dependent PRV accumulation in CAD neurites. (**A**) Differentiated CAD neurons were infected with P or ΔgE/gI PRV, then incubated for 24 h in the presence of 5 µM tubacin or DMSO solvent control (conditions are indicated at left of each row). Cells were then fixed and imaged in bright field or for mCherry-capsid fluorescence. Cell body fluorescence is deliberately overexposed in order to image particles in neurites. Two representative pairs of bright field and fluorescence images are shown in each row. Regions boxed in broken yellow lines are magnified 5× at the right of each row, with those from the left and right fluorescence panels depicted at the top and bottom, respectively. Scale bars are 10and 5 µm, as indicated. (**B**) Differentiated CAD neurons were infected with P PRV and incubated with 5 µM tubacin, 125 nM TSA or DMSO for 24 h then whole cell extracts prepared and western blotted for acetylated (AC)-α-tubulin or total α-tubulin, as indicated at left. (**C**) Images similar to those in (**A**) were used to count numbers of individual capsid-associated viral particle puncta (other than those in clusters) present in infected neurites in tubacin- or DMSO-treated CAD neurons. Plotted values are mean and SD from the mean for numbers of viral puncta counted in the neurites of 34 P-DMSO, 21 P-tubacin, 40 ΔgE/gI-DMSO, and 53 ΔgE/gI-tubacin treated CAD neurons.

We next tested whether the PRV-associated kinesin-1 motors play a functional role in transport of viral particles in the axon. Kinesin-1 motors preferentially bind to and traffic along microtubules that are stabilized and bundled as a consequence of α-tubulin acetylation (45–47). This makes it possible to use a pharmacological approach described by the Verhey laboratory (47) to test whether PRV particles employ kinesin-1 motors while trafficking within the axon. Normally, in differentiated CAD neurons, kinesin-1 cargo, such as JNK-interacting protein 1 (JIP1), accumulates only at the tip of a small subset of “axon-like” neurites containing elevated levels of acetylated α-tubulin and that usually corresponds to the longest neurite projecting from the cell body (47). Treatment of CAD neurons with the class I and II histone deacetylase (HDAC) inhibitor trichostatin A (TSA) (65), or the HDAC6 inhibitor tubacin (66), increases microtubule acetylation in the majority of neurites, with a concomitant enhancement of kinesin-1 transport resulting in the accumulation of JIP1 at nearly all neurite tips (47). We found that stimulation of microtubule acetylation had similar consequences for PRV trafficking. The addition of TSA to PRV-infected, differentiated CAD neurons elevated the levels of α-tubulin acetylation without affecting overall levels of α-tubulin expression (Fig. 6A) and increased the number of PRV particles in neurites, which often accumulated in large masses that we term clusters (Fig. 6B and C). Viral particle clusters accumulated at the tips of neurites and along their lengths (Fig. 6B), and one or more such clusters were found in ~45% of TSA-treated PRV-infected CAD neurons (Fig. 6D). Similar results were seen upon incubation of PRV-infected differentiated CAD neurons with tubacin. Tubacin treatment significantly increased the numbers of individual PRV puncta in neurites (Fig. 7A and C), the accumulation of acetylated α-tubulin (Fig. 7B), and the appearance of large clusters of viral particles within neurites (Fig. 7A). These data are consistent with kinesin-1 actively transporting PRV particles in the neurites of differentiated CAD neurons.

**Fig 8:**
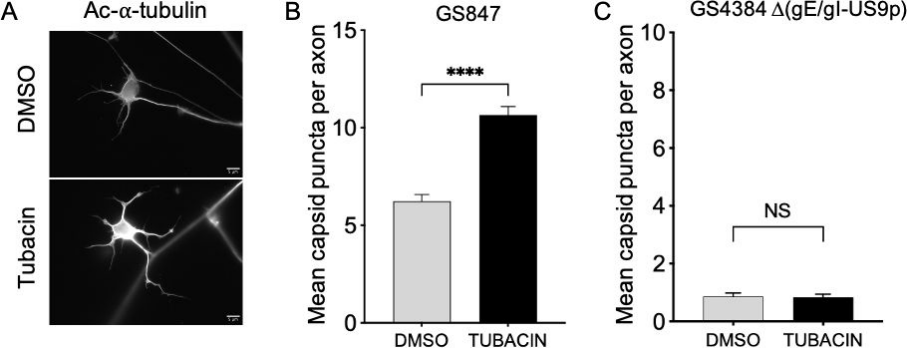
Tubacin stimulates microtubule acetylation and gE/gI-dependent PRV accumulation in the axons of DRG-derived sensory neurons. DRG-derived neurons were infected with GS847 then treated with DMSO or 5 mM tubacin exactly as in Fig. 7. (**A**) Cells were fixed, permeabilized, and immunostained for acetylated α-tubulin. Ac-α-tubulin levels are elevated in tubacin treated neurons. Scale bar: 10 µm. (**B**) Numbers of fluorescent GS847 puncta present in the axons of tubacin, or DMSO-treated, DRG neurons. Plotted values are the mean and SD from the mean for 120 and 113 axons (tubacin- and DMSO-treated, respectively). (**C**) Similar to (**B**) but for PRV-GS4384 [Δ(gE/gI-US9p)]. Plotted values are the mean and SD from the mean for 61 and 73 axons (tubacin- and DMSO-treated, respectively).

If the gE/gI complex is needed for recruitment of kinesin-1 motors (Fig. 3 and 4) then, in the absence of gE/gI, axonal PRV should fail to recruit kinesin-1 and no longer traffic as kinesin-1 cargo. In cultured neurons, PRV and HSV-1 mutants lacking gE/gI or US9p show dramatically reduced anterograde transmission; however, the phenotype is not absolute (1, 2, 28, 32, 39), and the numbers and trafficking properties of the rare PRV and HSV-1 particles that reach the axon can be measured (7, 28, 37, 44). Differentiated CAD neurons were infected with the PRV gE/gI-null mutant that is deficient for KIF5A/C, but not KIF1A, recruitment (Fig. 3). As expected, the number of gE/gI-null particles localized to neurites was dramatically reduced compared to the parental control (Fig. 7A and C). Strikingly, the residual population of gE/gI-null particles in neurites did not respond to microtubule hyperacetylation following tubacin addition (Fig. 7C). Thus, unlike parental PRV particles in CAD neurites, gE/gI-null particles did not behave in a manner typical of kinesin-1-driven cargo. We performed similar experiments in PRV-infected DRG-derived primary neurons. Tubacin treatment stimulated the levels of α-tubulin acetylation in infected neurons (Fig. 8A) and significantly increased the numbers of PRV puncta in axons (Fig. 8B). We do not have a GS847-derived PRV strain that lacks expression of only the gE/gI heterodimer, but simultaneous loss of gE/gI and US9p dramatically reduced the numbers of viral particles in sensory neuron axons, and this residual population did not respond to microtubule hyperacetylation (Fig. 8C). Thus, like gE/gI-null particles in CAD neurites, gE/gI-US9p-null virions in DRG neurons have lost the ability to traffic in a manner characteristic of kinesin-1-cargo. Taken together, our data are consistent with gE/gI-recruited KIF5A or KIF5C motors driving PRV traffic within the axons of CAD neural cells and DRG-derived neurons.

## DISCUSSION

### PRV prefers specific kinesin-1 isoforms in differentiated and undifferentiated CAD cells and in DRG-derived sensory neurons

We previously demonstrated that the PRV gE/gI-US9p complex recruits the neuron-specific kinesin-3 motor KIF1A, or the neuron-specific kinesin-1 isoform KIF5C, to distinct populations of egressing viral particles in differentiated CAD neural cells (20). In this study, we have further characterized kinesin-1 isoform selection in CAD cells and primary sensory neurons and tested the consequences of individual deletion of gE/gI or US9 for kinesin selection and axonal transport.

We found that PRV associates with all three KIF5 isoforms in CAD cells, but the selectivity of isoform recruitment is imposed by the state of CAD cell differentiation. Specifically, KIF5B became associated with PRV particles egressing from undifferentiated CAD cells, while KIF5A and KIF5C (20) were recruited to PRV particles egressing from differentiated CAD neurons (summarized in Fig. 2D). All three isoforms of KIF5 were expressed equally in CAD cells regardless of the differentiation state ([20] and Fig. 1 and 2). Neither KIF5A nor KIF5C were frequently colocalized with KIF1A on egressing particles prepared from differentiated CAD neurons, consistent with the hypothesis that kinesin-1 and kinesin-3 motors contribute to distinct stages of PRV egress. It remains to be determined whether the kinesin-1 KIF5A and KIF5C isoforms bind to the same, or distinct populations of egressing particles. Consistent with these data, PRV particles in the axons of infected DRG-derived sensory neurons are associated with KIF5C and to a much lesser extent with KIF5B or KIF1A. We have not yet tested the association of PRV with KIF5A in sensory neurons and cannot exclude the possibility that PRV may be associated with KIF5B in cellular locations other than the axon.

Why PRV prefers neuronal-specific KIF5A or KIF5C, rather than KIF5B, in differentiated CAD cells and DRG sensory neurons is unclear. However, there is evidence that these isoforms can have distinct transport functions in the nervous system. The axonal transport of mitochondria in zebrafish peripheral sensory neurons depends exclusively upon KIF5A (67), and only KIF5A dysfunction leads to seizure and Hereditary Spastic Paraplegia (59, 68). Moreover KIF5B, but not KIF5A, is involved in the development of excitatory synapses in postsynaptic neurons and transport of the RNA-binding protein Fragile X Mental Retardation Protein (FMRP) in dendrites (57, 69). PRV could select particular isoforms of KIF5 on the basis of isoform-specific post translational modifications or by discriminating between their divergent carboxy-terminal amino acid sequences (57). Alternatively, isoform selection might simply reflect the relative abundance of each type of kinesin-1 motor at the intracellular location where it becomes bound by PRV.

### US9p is important for kinesin-3 recruitment and enhances kinesin-1 recruitment

We earlier showed that loss of the entire gE/gI-US9p complex reduced the binding of KIF1A, and unexpectedly also KIF5C, from egressing PRV particles (20). In the current study, we demonstrate that the removal of US9p alone was sufficient to recapitulate this phenotype and that it applied to all three isoforms of KIF5. US9p-dependent recruitment of KIF1A to the viral particle is consistent with biochemical data demonstrating interaction between US9p and KIF1A in extracts of PRV-infected cells (21, 40, 41), but loss of US9p was not expected to impact KIF5 recruitment. One explanation is that US9p provides a receptor function for both KIF1A and KIF5 kinesins, and indeed HSV-1 US9p was reported to bind the C-terminal portion of KIF5B in pull-down assays using purified proteins (55). However, HSV-1 US9p has not been shown to interact with KIF5 in the context of a viral infection, and PRV US9p immunopurified from PRV-infected PC12 cells is only found in complex with KIF1A, not KIF5 (40). Furthermore, while HSV-1 particles were proposed to utilize KIF5 motors for transport within the axon (22), loss of US9p only diminishes the numbers of HSV-1 and PRV particles able to enter axons and not their ability to traffic along them (7, 37, 44). Thus, it is likely that KIF5 recruitment is mediated by factors other than US9p. For these reasons, we favor a model (Fig. 3D) in which US9p-dependent KIF1A transport (21, 40, 41) indirectly supports KIF5 recruitment by delivering PRV particles to a site, such as the proximal axon (47–51) where they then become associated with KIF5 motors (20). This model is consistent with the finding that KIF5C, and less frequently KIF1A, are found in association with PRV in the axons of primary sensory neurons (Fig. 5).

### The gE/gI complex is required for recruitment of kinesin-1 motors during egress

Removal of the gE/gI heterodimer resulted in loss of all KIF5 isoforms from egressing PRV particles (Fig. 3 and 4). US9p remained competent to bind KIF1A in the absence of gE/gI, consistent with *in vitro* data showing that gE/gI was absent from the US9p-KIF1A complex (21), though it does indirectly promote its assembly (41). The simplest explanation for our data is that the gE/gI heterodimer is required for the attachment of kinesin-1 motors to the egressing PRV particle (summarized in Fig. 3D). This is consistent with our finding that PRV particles isolated from porcine kidney PK15 epithelial cells demonstrate reduced kinesin-dependent motion along microtubules *in vitro* when gE/gI-US9p are absent (20); PK15 cells lack the neuron-specific KIF1A motor (16, 19) and US9p-null PRV mutants exhibit no apparent phenotype in these cells (29), so it is most likely that the motility defect of PK15-derived gE/gI-US9p null particles results from failure to recruit KIF5B via gE/gI. A role for gE/gI in KIF5B assembly onto egressing particles would also help explain the importance of the gE/gI complex in sorting of alphaherpesviruses to the basolateral surfaces of polarized epithelial cells (25, 70–73).

The finding that US9p is dispensable for PRV and HSV-1 trafficking within axons provided an initial indication that anterograde axonal transport utilizes a second kinesin recruitment mechanism (2, 7, 15, 37, 44). This hypothesis was supported by the detection of kinesin-1 on egressing HSV-1 particles in the axons of primary neurons (52) and the axon-like neurites of differentiated CAD cells (22). Moreover, silencing of KIF5 antagonized HSV-1 anterograde transport in CAD neurons, while silencing of KIF1A had little effect (22). In the present study, the importance of KIF5 for PRV transport within axons, and of gE/gI as a KIF5 effector, was further validated by selective stimulation of KIF5 function as a consequence of microtubule hyperacetylation. Kinesin-1, but not kinesin-3 motors such as KIF1A, preferentially traffic along subsets of axonal microtubules that are stabilized and bundled as a result of acetylation at α-tubulin residue K_40_ (45–47). Acetylation-driven microtubule bundling enhances kinesin-1 run lengths and increases the number of available kinesin-1 binding sites (46). As previously reported for endogenous kinesin-1 cargoes (47), incubation of differentiated CAD neurons with TSA (65) or tubacin (66) increased the numbers of viral particles at neurite tips and along neurite shafts (Fig. 6 and 7). Tubacin similarly stimulated the numbers of PRV particles present in the axons of primary sensory neurons (Fig. 8). The observation that PRV particles in CAD cell neurites and sensory neuron axons respond to microtubule hyperacetylation in this manner argues that they are transported as kinesin-1 cargo. The elevated numbers of axonal viral particles may reflect their enhanced kinesin-1-mediated transport from the site at which KIF5A/C motors are attached after the virions enter the axon from the soma. If KIF5A/C-attachment occurs very close to the cell body, or at the cell body/axon junction, our fluorescence imaging studies might be unable to resolve these virions from the bright fluorescence of the infected cell body. In addition, it is known that a subpopulation of PRV particles enters axons and exhibits apparently wild-type anterograde transport even in the absence of US9p (see Introduction and reference [44]). If this finding means that some PRV particles can utilize kinesin-1 to enter axons then the efficiency of this axonal delivery and their subsequent axonal trafficking would presumably both be enhanced by microtubule hyperacetylation. In either case, removal of the gE/gI complex rendered axonal PRV particles insensitive to microtubule hyperacetylation (Fig. 7 and 8), suggesting that kinesin-1-mediated transport of PRV particles is gE/gI dependent.

### Utilization of kinesin-1 and kinesin-3 motors by PRV resembles trafficking strategies of cellular cargo in uninfected neurons

**Fig 9:**
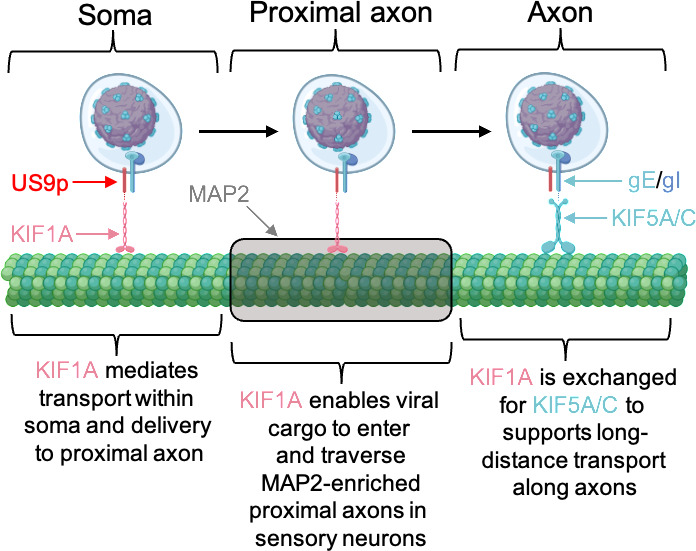
Model for gE/gI-US9p-mediated kinesin recruitment during egress from sensory neurons. Schematic to explain and summarize our data. Egressing virus particles, the gE/gI-US9p complex, and kinesins are represented as in Fig. 3D. Microtubules are shown in green. PRV utilizes US9p to recruit KIF1A for traffic into the proximal axon, then at some later point exchanges KIF1A for KIF5A or KIF5C, using a gE/gI-dependent mechanism. KIF1A may be required for early stages of axonal traffic in sensory neurons because a high concentration of MAP2 in the proximal axon (gray rectangle) prevents kinesin-1 motors from associating with microtubules. See Discussion for more details.

Combining our model for the role of the gE/gI-US9p complex in kinesin recruitment (Fig. 3D) with our data from infected primary explanted neurons (Fig. 5) and the effects of microtubule hyperacetylation (Fig. 6 to 8), we propose a model in Fig. 9 to summarize and explain our data. We suggest that in the soma, gE/gI-US9p recruits KIF1A to egressing PRV particles via US9p, then delivers those particles to another location (such as the proximal axon in sensory neurons [47–51]), where gE/gI recruit KIF5A/C motors for long-distance anterograde transport along the axon. This model allows for crosstalk between the KIF1A and KIF5 recruitment machinery, enabling US9p and gE/gI to spatially and temporally engage distinct kinesin motors while the virus navigates the complex intracellular environment of the neuron. Such a mechanism finds precedent in the traffic of normal neuronal cargo, where cooperation between kinesin-1 and kinesin-3 motors is a general strategy for controlling transport between the cell body and axon (74). During movement of DCVs (dense core vesicles) from the soma to the axon in rodent DRG neurons, a high concentration of soluble MAP2 (microtubule-associated protein 2) in the proximal axon inhibits microtubule-binding by kinesin-1, requiring DCVs to select kinesin-3 motors for transport into the axon (75). When trafficking DCVs reach the distal axon, they encounter diminished levels of MAP2, and kinesin-1 motors then become able to support long-range transport along the axon (75, 76). Similar coordination is seen in the motor neurons of Drosophila larvae, where DCVs are transported from the cell body into the axon by the kinesin-3 motor Unc-104, after which Unc-104 cooperates with kinesin-1 to support fast, highly processive runs toward axon termini (77). Conversely, in rat hippocampal neurons, TrkB (tropomyosin-related kinase receptor B)-carrier vesicles require kinesin-1 (KIF5C) for their formation and delivery to the proximal axon, but subsequent transport to the distal axon depends upon KIF1A (74). We note that, in these examples, it is thought to be the activity of cargo-bound kinesin-1 or kinesin-3 that changes during transport, whereas we propose that gE/gI-US9p regulates PRV association with one or the other motor during egress. Interestingly, gE/gI-US9p promotes proteosomal degradation of KIF1A within the environment of the axon (78), and we speculate that this could facilitate replacement of bound KIF1A by KIF5 on the egressing viral particle.

Our understanding of how, and why, cellular cargoes select particular kinesins, or combinations of kinesins, for their transport within neurons and other cell types remains quite limited (17–19, 75, 79). The gE/gI-US9p complex demonstrates one mechanism by which kinesin KIF1A or specific isoforms of KIF5 might be selected, depending upon the stage of egress and the differentiation state of the host cell.

## MATERIALS AND METHODS

### Cell lines and viruses

CAD cells were grown and differentiated as previously described (20). For expression of the mCitrine-KIF1A fusion protein, undifferentiated CAD cells were transfected with the appropriate expression construct using Lipofectamine 3000 (Life Technologies) according to the manufacturer’s protocol, as previously described (20). After 3 days, the transfected CAD cells were either infected with PRV or were transferred to a differentiation medium for 5 days prior to infection. PK15 cells were cultured as previously described (20). Vero cells were maintained in Dulbecco’s Modified Eagle Medium (Gibco Laboratories) supplemented with 10% newborn calf serum (NCS, Peak Serum Inc.) and 1% penicillin-streptomycin (Gibco Laboratories).

PRV virus stocks were prepared by low multiplicity infection of PK15 cells as previously described (20, 80) and titers determined on Vero cells. PRV infection of undifferentiated CAD cells or differentiated CAD neurons for subcellular fractionation analysis was as previously described (20). For experiments in which viral particles were imaged and counted in intact CAD cell neurites, differentiated CAD neurons were infected with Benzonase nuclease (Sigma Aldrich)-treated PRV stocks prepared from the culture medium of infected PK15 cells, as described in our earlier studies with HSV-1 (12).

PRV strain GS4284 is wild type, other than encoding an mCherry fluorescent protein fused in frame with the UL25p capsid protein, as previously described (20, 56, 81, 82). Mutants derived from PRV-GS4284 were constructed by two-step RED-mediated recombination using a derivative of the pBecker3 infectious clone, pGS4284. PRV-GS5469 encodes a deletion in the US9 gene, as previously described (44). For the construction of PRV-GS7453, deleted for genes US7 and US8, the following primer pair were made with 5' homologies to sequences upstream of US7 and downstream of US8, and 3' homologies (underlined) to pEP-KanS2 (83):

5'AAAAGCGCAGCCCGGTCCGTAGCCTCCGCAGTACCGGCGTCGATG**TAA**CTCTCGCCGGTGTACAGGATGACGACGATAAGTAGGG and

5'GTAGTAGTCCTCGTGCGTGGGCAGGCTGGTGTACACCGGCGAGAG**TTA**CATCGACGCCGGTACCAGTGTGATGGATATCTAGG. The primers encode a single deletion that removes US7 and the first 473 codons of US8. The remaining US8 coding sequence contains no in-frame ATG start codons, and a TAA stop codon was placed at the deletion junction in-frame with the remaining US8 coding sequence to prevent spurious upstream translation initiation. This design preserves the last 315 nucleotides of the US8 coding sequence to avoid disruption of the promoter that drives downstream US9 expression. The PCR product was recombined into the pGS4284 clone harbored in the GS1783 recombineering *Escherichia coli* strain for the two-step recombination procedure (82). The resulting recombinant was given the designation pGS7453. Transfection of pGS7453 into PK15 cells was as previously described (84) and yielded an initial viral stock of PRV-GS7453. Working stocks of PRV-GS7453 were made by additional passage through PK15 cells.

PRV strain GS847, derived from a pBecker3 infectious clone, is wildtype other than encoding an mRFP1 fluorescent protein fused in frame with the UL35p capsid protein and has been previously described (64). PRV-GS4384 is identical to PRV-GS847 except for deletion of genes US7, US8, and US9. It was constructed in a manner similar to that described above for PRV-GS7453 using the primers:

5'GGTCCCGCGGACACCGACGAGCTAAAAGCGCAGCCCGGTCCGTAGTCGCTGTCGGCGCTGAGGATGACGACGATAAGTAGGG and

5'CTACACGTGCCGGGCGATGATGCCCCCGATCAGCGCCGACAGCGACTACGGACCGGGCTGCAACCAATTAACCAATTCTGATTAG.

### Isolation and culture of embryonic rat DRG-derived sensory neurons

DRG sensory neurons were prepared from embryonic day 16 rats as previously described (85). All experiments complied with all relevant ethical regulations for animal testing and research and were approved by the Albert Einstein College of Medicine Institutional Animal Care and Use Committee (IACUC, IACUC protocol number 0001209). Rats were housed and handled at the Albert Einstein College of Medicine. Euthanasia was done with a lethal dose of CO_2_ gas delivered by compressed CO_2_ canisters using the recommended gas flow rate prior to decapitation. The Panel on Euthanasia of the American Veterinary Medical Association recommends these procedures.

### Construction and use of a recombinant lentivirus expressing mCitrine-KIF1A

The gene encoding an mCitrine-KIF1A fusion has been previously described (60, 61) and used in our earlier studies (20). It was amplified by PCR using the primers: 5'CTCAAGCTTCGAATTCCTCGAGCTCAAGCTTATGGCT and

5'TAGAGTCGCGGGATCCTTATTACTTGTACAGCTCGTCCATG. The PCR product was inserted into the lentiviral expression vector pLVX-Puro (Takara, Cat# 632164) between restriction sites EcoRI and BamHI by In-Fusion HD Cloning (Takara, Cat# 638910). To reduce the size of the expression vector and ensure efficient lentiviral packaging, the vector was amplified by PCR using the primers 5'ATTCTACCCCGCGTCTGGAACAATCAACC and

5'GACGCGGGGTAGAATTATCTAGAGTCGCGGG, then recircularized by In-Fusion HD Cloning, resulting in removal of the puromycin resistance gene and its promoter. The resulting lentivirus expression backbone was then transfected into the Lenti-X 293T Cell Line (Takara, Cat# 632180) using the Lenti-X Packaging Single Shot protocol (Takara, Cat# 631278). Lentivirus particles were harvested at 48 h post transfection from the cell supernatant and titrated using Lenti-XGoStix Plus (Takara, Cat# 631280).

### Fluorescence imaging, immunostaining, and data analysis

Undifferentiated CAD cells or differentiated CAD neurons were infected with PRV strains GS4284, GS5469, or GS7453 as previously described (20), and the infected cells, or subcellular fractions prepared from them, fixed, immunostained, and imaged as follows.

CAD cell PNSs were prepared and subjected to density gradient centrifugation, and the resulting membrane-associated buoyant float-up virus fractions were attached to poly-L-lysine (Sigma-Aldrich)-coated glass coverslips as previously described (20). They were then fixed with 4% paraformaldehyde in phosphate buffered saline (PBS) for 30 min at room temperature (RT) and washed with PBS. For direct fluorescence imaging, coverslips were immediately mounted using ProLong Diamond Antifade agent (Life Technologies, Cat# P36961). For immunostaining, coverslips were incubated in blocking buffer (2 mg/mL bovine serum albumin in PBS) for 30 min. For detection of isoforms of KIF5, coverslips were incubated for 1 h at RT with rabbit antibodies specific for KIF5A (Thermo Fisher Scientific, Cat# PA1-642), KIF5B (Thermo Fisher Scientific, Cat# PA1-643), or KIF5C (abcam, Cat# ab 193352), or with an equal mass of control rabbit IgG (Sigma-Aldrich, Cat# I5006). Samples were then washed with blocking buffer and incubated with Alexa Fluor 405-labeled goat anti-rabbit IgG (Life Technologies, Cat# A31556) for 1 h at RT and then washed and mounted for imaging as described previously (20). Images were acquired using an Inverted Olympus IX81 microscope with a 60× oil-immersion, 1.4-numerical aperture (NA) objective. All images were saved in TIF format using IP Lab 4.0.8 software. Colocalization of fluorescence signals was determined by intersecting analysis using Volocity Quantitation software (Quorum Technologies, Inc.).

Intact, infected cultured CAD cells were imaged as described above, and NIH-ImageJ software was used for image analysis. Virus particle numbers in the neurites of differentiated CAD neurons were counted using the open source software Fiji (http://fiji.sc) (86, 87). Numbers of capsid-associated puncta were determined in the first 25 µm of the neurite projecting from the cell body and straightened using the Fiji “Edit > Selection > Straighten” option.

Explanted DRG-derived neurons were cultured on poly-D-lysine (Millipore Sigma, Cat# P6407) and laminin-treated coverslips or μ-slides (ibidi, Cat #80800, #80281) as previously described (85), prior to infection by PRV. In some experiments, neurons were infected with a recombinant lentivirus, designed to express mCitrine-KIF1A, 24 h prior to infection by PRV. Unless otherwise specified, neurons were fixed after 18 h of PRV infection using 4% paraformaldehyde in PBS for 20 min at RT, washed with PBS, permeabilized with 1% Triton X-100 in PBS for 10 min at RT, and blocked with 10% NCS and 0.1% Triton X-100 in PBS for 0.5 h at RT. Immunostaining for isoforms of KIF5 was performed using the same primary antibodies as described above and Alexa Fluor 647-labeled goat anti-rabbit IgG (Life Technologies, Cat# A21245). Samples prepared on coverslips were mounted using ProLong Diamond Antifade agent as described above. Samples prepared on μ-slides were overlaid with a minimum volume of PBS, prior to imaging. Images were captured with a Nikon ECLIPSE Ti-E inverted microscope fitted with a 60× oil-immersion/1.4 NA objective, coupled to a CSU-W1 confocal head (Yokogawa Electric Corporation) and ORCA-FLASH 4.0 sCMOS camera (Hamamatsu Photonics). Images were saved in Nikon ND2 file format using NIS-Elements Viewer Imaging software, and analyses were performed using the Bio-Formats Importer plugin to open ND2 files. Puncta were counted using 32 µm of axon straightened using the Fiji “Edit > Selection > Straighten” option.

The integrated density of mCitrine-KIF1A fluorescence, as well as anti-KIF5B and anti-KIF5C immunofluorescence, was measured in PRV-infected DRG soma and a 23 μm length of proximal axon using the ImageJ Analyze >Measure tool. The background mean fluorescence value was multiplied by the soma or axon area then subtracted from the integrated fluorescence density data.

In all imaging experiments, whether of cell extracts or whole cells, levels of non-specific background fluorescence were determined from appropriate negative control samples. For example, this could be achieved by imaging or preparing extracts from cells that had not been transfected to express mCitrine-KIF1A, or by replacing a primary antibody by an equivalent mass of an unrelated control antibody. Negative control samples were always imaged side-by-side with experimental samples and used to select imaging conditions under which the negative control samples appeared black.

### Western blotting

Whole cell extracts were subjected to SDS-PAGE electrophoresis on 7.5% gels then transferred to a polyvinylidene difluoride membrane as previously described (88), blocked using a solution of 5% dried milk in Tris-buffered saline tween (TBST; 137 mM NaCl, 0.1% [wt/vol] Tween-20, and 20 mM Tris.Cl pH 7.4), and then incubated with the appropriate primary antibody overnight at 4°C. Anti-KIF5A, -KIF5B, and -KIF5C antibodies were identical to those used for immunostaining (see above). Antibodies to α-tubulin (Millipore Sigma, Cat# T9026), acetylated-α-tubulin (Millipore Sigma, Cat# T7451), and β-Actin (Santa Cruz Biotechnology Inc., Cat# sc47778) were used at dilutions as per suppliers’ recommendation. After washing, membranes were incubated with peroxidase-conjugated anti-rabbit or anti-mouse secondary antibodies (Millipore Sigma) and bound antibody detected with an enhanced chemiluminescence (ECL) substrate (PerkinElmer).

## Data Availability

The quantitation of image data used to generate the figures in this article has been deposited in figshare: https://figshare.com/articles/dataset/JVI_Datasets_2024_pzfx/27337152?file=50083497.
